# Isolation and genome sequencing of *Staphylococcus schleiferi* subspecies *coagulans* from Antarctic and North Sea seals

**DOI:** 10.1099/acmi.0.000162

**Published:** 2020-08-21

**Authors:** Geoff Foster, Andrew Robb, Gavin K. Paterson

**Affiliations:** ^1^​ SRUC Veterinary Services, Inverness IV2 5NA, UK; ^2^​ Scottish MRSA Reference Laboratory, Glasgow RG6 6BZ, UK; ^3^​ Royal Dick School of Veterinary Studies and The Roslin Institute, University of Edinburgh, Edinburgh EH25 9RG, UK

**Keywords:** bacterial genomics, coagulase-positive staphylococci, otariid, seals, *Staphylococcus schleiferi*

## Abstract

Reports on the commensal organism and opportunistic pathogen *Staphylococcus schleiferi* have largely considered isolates from humans and companion dogs. Two subspecies are recognized: the coagulase-negative *S. schleiferi* ssp. *schleiferi*, typically seen in humans, and the coagulase-positive *S. schleiferi* ssp. *coagulans*, typically seen in dogs. In this study, we report the isolation, genome sequencing and comparative genomics of three *S. schleiferi* ssp. *coagulans* isolates from mouth samples from two species of healthy, free-living Antarctic seals, southern elephant seals (*Mirounga leonina*) and Antarctic fur seals (*Arctocephalus gazella*), in the South Orkney Islands, Antarctica, and three isolates from post-mortem samples from grey seals (*Halichoerus grypus*) in Scotland, UK. This is the first report of *S. schleiferi* ssp. *coagulans* isolation from Antarctic fur seal and grey seal. The Antarctic fur seal represents the first isolation of *S. schleiferi* ssp. *coagulans* from the family *Otariidae*, while the grey seal represents the first isolation from a pinniped in the Northern Hemisphere. We compare seal, dog and human isolates from both *S. schleiferi* subspecies in the first genome-based phylogenetic analysis of the species.

## Introduction

*Staphylococcus schleiferi* appears primarily to be a commensal and opportunistic pathogen of humans and domestic dogs. Two subspecies are recognized, *S. schleiferi* ssp. *schleiferi* [1] and *S. schleiferi* ssp. *coagulans* [2]. *S. schleiferi* ssp. *coagulans* is identified phenotypically on the basis of free coagulase production (tube test), urease production and the ability to ferment ribose, which are properties that *S. schleiferi* ssp. *schleiferi* typically lacks [2]. The two subspecies are also differentiated based on DNA–DNA hybridization [2] and matrix-assisted laser desorption/ionization time-of-ﬂight mass spectrometry [3]. However, not all studies differentiate the two subspecies and some authors have highlighted potential difficulties with their separation [4, 5]. Both subspecies can encode methicillin resistance, as well as other antimicrobial resistance, heightening their potential clinical significance [6–9].

*S. schleiferi* ssp. *schleiferi* can be isolated from human preaxillary skin [10, 11] and nares [12] and is associated with a range of nosocomial infections, including urinary tract infections [13], brain abscess and cerebrospinal fluid culture [14], pacemaker- and catheter-related infections [10, 14], surgical wound infections [14, 15] and endocarditis [16]. In contrast, *S. schleiferi* ssp. *coagulans* is rarely found in humans [5], but is frequently isolated from healthy dogs from the skin [17] and the external ear canal [18], as well as being associated with external ear otitis [2, 18–20] and pyoderma [19, 21, 22]. *S. schleiferi* ssp. *schleiferi* has also been reported from dogs [19] and both subspecies have been isolated from cats [23]. In addition, *S. schleiferi* ssp. *coagulans* has been reported from chicken meat [24], ready-to-eat retail fish [25] and the posterior nares and cloaca of healthy feral and domestic pigeons (family *Columbidae*) [26]. *S. schleiferi *, not delineated to subspecies level, has been isolated from clinical material in farmed mink [27]. Finally, *S. schleiferi* ssp. *coagulans* has been isolated in Antarctica from the cloaca and beaks of Adélie penguins (*Pygoscelis adeliae*), from South Polar skua (*Stercorarius maccormicki*) droppings, and the anus of two species of seals from the family *Phocidae*: Weddell seals (*Leptonychotes weddellii*) and southern elephant seals (*Mirounga leonina*) [28]. Thus the host range of *S. schleiferi* extends beyond humans and dogs and likely includes a range of hitherto unreported host species.

In this study, we report the isolation and genome sequencing of three *S. schleiferi* ssp. *coagulans* isolates from three Antarctic seals; two southern elephant seals (*M. leonina*) and an Antarctic fur seal (*Arctocephalus gazella*) and three isolates from grey seals (*Halichoerus grypus*) in the North Sea. To the best of our knowledge, isolates from these three host species have not been genome sequenced previously and this report represents the first isolation of *S. schleiferi* from a member of the family *Otaridae*, an Antarctic fur seal (*A. gazella*) and the first from grey seal (*H. grypus*). To place these seal isolates into the context of the *S. schleiferi* population we present the first genome-based phylogenetic analysis of the species comparing both subspecies and including isolates from humans, dogs and three seal species.

## Material and methods

### Bacterial isolation

Healthy, free-living Antarctic seals were sampled by the British Antarctic Survey at two separate sites on Signy Island in the South Orkney Islands, Antarctica in 1993. A swab on the end of a broomstick was used to collect an oral sample when a male (territorial) seal yawned. The samples were freeze-dried and subsequently examined by microbiological culture at Scotland’s Rural College (SRUC) Veterinary Services, Inverness. Three further isolates were collected post-mortem from three grey seals found dead on the North Sea shoreline in Fife, eastern Scotland between 2002 and 2016 and reported under the Scottish Marine Animals Strandings Scheme (SMASS). Carcases were transported to SRUC Veterinary Services, Inverness for a post-mortem examination and selected tissues and gross lesions were sampled for microbiological and histopathological diagnoses. The animals likely died from phocine distemper virus, emphysema and necrotizing haemorrhagic gastro-enteritis, respectively. *S. schleiferi* ssp. *coagulans* was isolated from several organs from these grey seals; the isolates subjected to further study here were collected from the lungs. Isolation was made on Columbia agar supplemented with 5 % sheep blood (CSBA) (Oxoid, Basingstoke, UK) incubated in air plus 5 % CO_2_ at 37 °C for 18–24 h. Sub-cultures were made to CSBA for characterization tests. Initial identification to species was made by API ID 32Staph (bioMérieux, Basingstoke, UK). Antimicrobial sensitivity testing was performed using Vitek 2 (bioMérieux, Basingstoke, UK) following the manufacturer’s instructions. Using the AST-P634 Staphylococcus card the antimicrobials tested were: cefoxitin screen, benylpenicillin, oxacillin, gentamicin, ciprofloxacin, inducible clindamycin resistance, erythromycin, clindamycin, linezolid, daptomycin, teicoplanin, vancomycin, tetracycline, nitrofurantoin, fuscidic acid, chloramphenicol, rifampicin and trimethorprim, with interpretation performed using the Clinical and Laboratory Standards Institute (CLSI) criteria (2015). Clumping factor and free coagulase were tested with lyophilized rabbit plasma with EDTA (Oxoid).

### Whole-genome sequencing

Whole-genome sequencing was performed by Microbes NG (University of Birmingham, UK) using Illumina technology with 2×250 bp paired-end reads. Genomic DNA libraries were prepared using the Nextera XT Library Prep kit (Illumina, San Diego, USA) following the manufacturer’s protocol with the following modifications: 2 ng of DNA instead of 1 were used as input, and the PCR elongation time was increased to 1 min from 30 s. Reads were trimmed using Trimmomatic version 0.30 [29] and a sliding window quality cut-off of 15. Genome assembly was performed *de novo* using SPAdes version 3.7, with default parameters for 250 bp Illumina reads [30], and annotated by the National Center for Biotechnology Information (NCBI) Prokaryotic Genome Annotation Pipeline [31].

### Genome analysis

Average nucleotide identity (ANI) was calculated using the EZBioCloud ANI Calculator (https://www.ezbiocloud.net/tools/ani) [32]. Acquired resistance genes were identified using ResFinder-3.1 employing the threshold of 60 % for percentage identity and minimum length of 60 % [33].

Phylogenetic relationships between study isolates and previously sequenced, assembled and annotated *S. schleiferi* isolates [34–37] was inferred using CSI Phylogeny 1.4 (Call SNPs and Infer Phylogeny) [38] with *S. schleiferi* ssp. *schleiferi* ATCC43808^T^ (GCA_900458895.1) as the reference genome and applying default settings [minimum depth at single-nucleotide polymorphism (SNP) positions: 10×; minimum relative depth at SNP positions: 10 %; minimum distance between SNPs (prune): 10 bp; minimum SNP quality: 30; minimum read mapping quality: 25; and minimum *Z* score: 1.96]. We found 2 115 918 positions in all analysed genomes. The resultant tree was annotated using the Interactive Tree of Life (iTOL) [39]. Two further, hitherto unpublished, *S. schleiferi* ssp. *coagulans* canine isolates from the Royal (Dick) School of Veterinary Studies were also included for comparison (Table S1, available in the online version of the article).

### Isolate and data availability

Isolates metadata and nucleotide accessions are provided in Table S1. The genome sequenced isolates A/G14/99/8, A/G16/00/1, A/W41/99/1 and M615/02/4 have been deposited with the Culture Collection University of Gothenburg as CCUG52137, CCUG52138, CCUG53690 and CCUG 52139, respectively.

## Results

### Isolation of Antarctic and North Sea seal *S. schleiferi*

In the course of British Antarctic Survey bacteriological investigations of mouth samples collected from seals at Signy Island, South Orkneys in Antarctica, four isolates of *S. schleiferi* were isolated from three animals. Isolates A/G14/99/8 and A/G16/00/1 and AG16/00/7 were recovered from two southern elephant seals (*M. leonina*) sampled at the Gourlay Peninsula at the south-easternmost end of the island and A/W41/99/1 was recovered from an Antarctic fur seal (*A. gazella*) sampled at the Wallows in the north-east. With isolates A/G16/00/1 and A/G16/00/7 having been isolated from the same animal, only A/G16/00/1 from these two was taken forward for further study. Isolates M615/02/4, M31/11/1, M611545/16/1 were collected post-mortem from the lungs of grey seals that had stranded in different parts of Fife (North Sea coast), Scotland in 2002, 2011 and 2016, respectively. The three North Sea isolates were tube coagulase- and urease-positive, clumping factor-negative and unable to ferment trehalose, phenotypes indicative of *S. schleiferi* ssp. *coagulans*. The three Antarctic seal isolates shared these features, with the exception that they were each able to ferment trehalose.

### Genome sequencing and genomic-based identification

To investigate these six isolates further they were genome sequenced using HiSeq technology. The genome sizes and GC % content were as follows: A/G14/99/8, 2 402 027 bp, 35.8 %; A/G16/00/1, 2 374 021 bp, 35.9 %; A/W41/99/1, 2 402 964 bp, 35.8 %; M615/02/4, 2 590 557 bp, 35.9 %; M31/11/1, 2 471 374 bp, 35.9 %; and M611545/16/1, 2 399 198 bp, 36.1 %. To confirm the identity of the six seal isolates to species and subspecies levels on the basis of genome sequence, a comparison with the two genome-sequenced *S. schleiferi* subspecies type strains (accessions in Table S1) was performed using ANI. In each case the ANI was closer to *S. schleiferi* ssp. *coagulans* (97.47 %–98.89 %) than to *S. schleiferi* ssp. *schleiferi* (95.09–95.68 %), consistent with all six seal isolates belonging to *S. schleiferi* ssp. *coagulans* (Table S1).

### Phylogenetic relationships among *S. schleiferi*

**Fig. 1.**
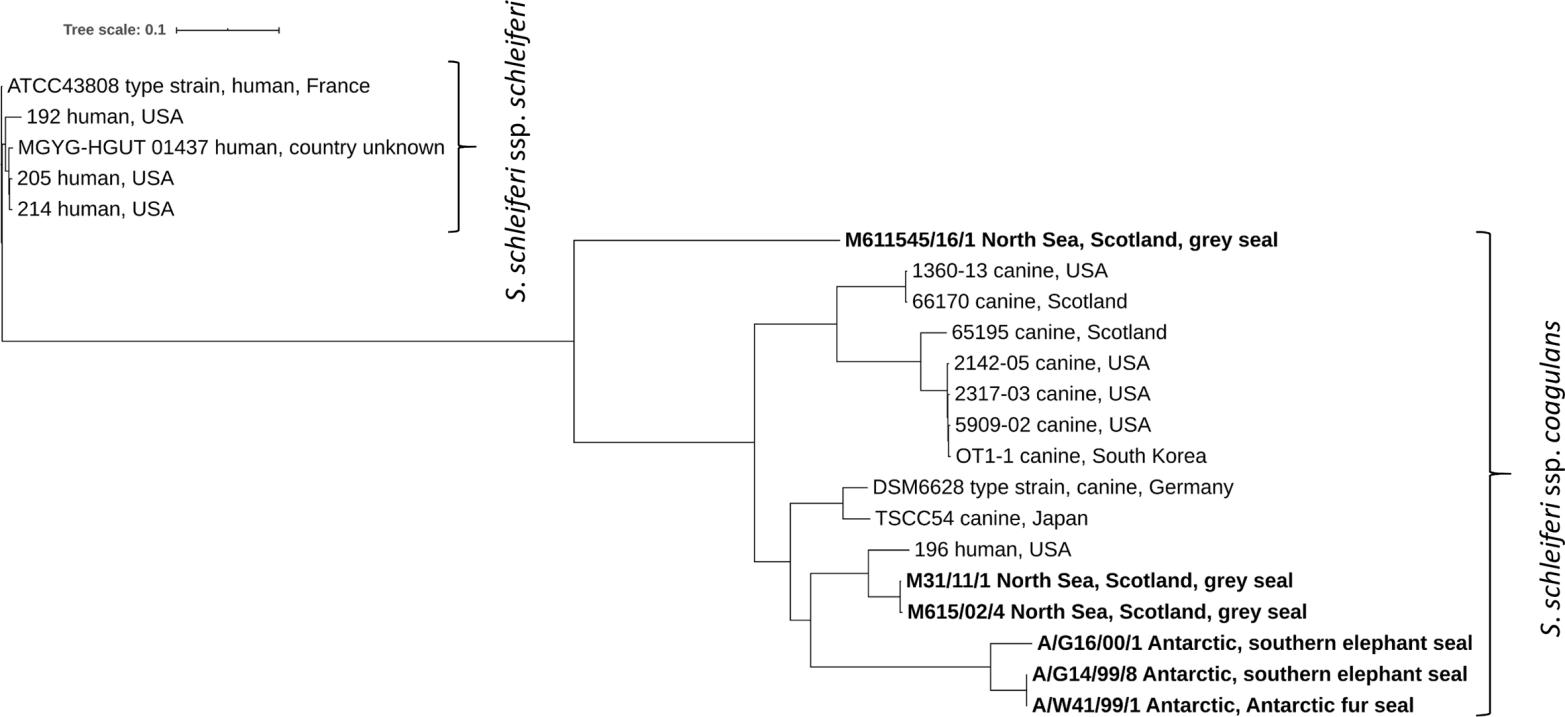
Phylogenetic tree of sequenced *S. schleiferi* isolates. Generated from SNPs across 2 093 618 positions in the core genome using CSI Phylogeny 1.4(38) with *S. schleiferi* ssp. *schleiferi* ATCC43808^T^ (GCA_002901995.1) as the reference genome and the tree root. Seal isolates from this study are highlighted in bold. Host and country of origin indicated, where known. Genome accessions are provided in Table S1. Subspecies assigned based on ANI to type strains of *S. schleiferi* ssp. *coagulans* (DSM6628^T^) and *S. schleiferi* ssp. *schleiferi* (ATCC43808^T^).

To compare the relationships between the six seal isolates and other genome-sequenced *S. schleiferi* isolates, a phylogenetic tree was constructed based on SNPs across the core genome (Fig. 1). The phylogeny clearly delineated the two *S. schleiferi* subspecies, with the six seal isolates belonging to *S. schleiferi* ssp. *coagulans*. The six seal isolates were split between three clades, indicating that diverse *S. schleiferi* ssp. *coagulans* lineages are present in seals. The Antarctic seal isolates A/G14/98/1 and A/W41/99/1 are identical and most closely related to the third Antarctic seal isolate, A/G16/00/1 (separated by 4289 SNPs). Likewise, the two North Sea grey seal isolates M31/11/1 and M615/02/4 are closely related, being separated by 150 SNPs. These two clusters of seal isolates are, however, distinct, being separated by at least 14 621 SNPs. In contrast, the final North Sea grey seal isolate (M611545/16/1) is not closely related to any other analysed isolate, with its closest relative differing by 22661 SNPs and the closest seal isolate being separated from it by 23 316 SNPs. There were two other closely related clusters of *S. schleiferi* ssp. *coagulans* isolates. Isolates 2142, 2317, 5909 and OT-1 are separated by a mean pairwise SNP difference of 187, while 1360 and 6124 are separated by 142 SNPs (Fig. 1).

### Antimicrobial resistance

None of the six seal isolates, A/G14/99/8, A/G16/00/1, A/W41/99/1, M615/02/4, M31/11/1and M611545/16/1, displayed phenotypic resistance against the antimicrobials tested. Consistent with that finding, no acquired antimicrobial resistance genes were identified in their genome sequences.

## Discussion

We describe here the isolation and whole-genome sequencing of six isolates of *S. schleiferi* ssp. *coagulans* from three species of pinniped, namely southern elephant seal, Antarctic fur seal and grey seal. While *S. schleiferi* ssp. *coagulans* has been isolated from Antarctic seals previously [28], those isolates have not been characterized further than identification using partial 16S rRNA gene sequencing and to the best of our knowledge this study represents the first isolation of *S. schleiferi* from Antarctic fur seal and grey seal. This finding enhances our understanding of the distribution of this commensal and opportunistic pathogen and extends the known host range of *S. schleiferi* ssp. *coagulans* to now include dogs, humans, feral and domestic pigeons, southern elephant seals, Antarctic fur seal, grey seals, Weddell seal, Adélie penguins, South Polar skua and possibly chicken and fish. This diverse range of host species suggests that *S. schleiferi* ssp. *coagulans* may have a broad host range comprising other as yet unrecognized host species. Veterinary diagnostic laboratories should therefore consider the possible diagnosis of *S. schleiferi* ssp. *coagulans* among coagulase-positive staphylococci isolated from any host species. In the case of the Antarctic seal isolates in this study, they were recovered from mouth swabs from apparently healthy animals, suggesting that *S. schleiferi* ssp. *coagulans* is an oral commensal in this setting. The grey seal isolates in this study were isolated post-mortem from dead seals with underlying diseases, but may also have carried commensal *S. schleiferi* ssp. *coagulans* that disseminated to the lungs and other organs during ill health or following death. While no link to infection is apparent from these current data, it would be reasonable, based on its epidemiology in dogs, to consider that *S. schleiferi* ssp. *coagulans* may also act as a commensal and opportunistic pathogen in seals and other hosts.

The three Antarctic seal isolates in this study were able to ferment trehalose, which is considered to be a feature of *S. schleiferi* ssp. *schleiferi* but not typical of *S. schleiferi* ssp. *coagulans* [2]. The isolates therefore represent further phenotypic diversity among *S. schleiferi* ssp. *coagulans* and highlight the potential difficulty of relying on any single phenotype to differentiate closely related bacterial species or subspecies.

The phylogenetic analysis clearly differentiated the two *S. schleiferi* subspecies, and in agreement with ANI data, showed that all six seal isolates belonged to the subspecies *S. schleiferi* ssp. *coagulans*. While some of the seal isolates were closely related to each other, distinct strains are nonetheless present in seal species. Interestingly, A/G14/99/8 and A/W41/99/1 were identical across all 2 093 618 positions, despite being isolated at different sites and being from different host species, which is suggestive of transmission between individual Antarctic seals or exposure to a common source.

While the number of sequenced isolates is small for both *S. schleiferi* subspecies, the phylogenetic analysis does indicate rather limited diversity among the *S. schleiferi* ssp. *schleiferi* isolates sequenced to date compared to *S. schleiferi* ssp. *coagulans* (mean pairwise SNP distance 816 versus 14 189). This may be partly caused by the wider range of host species and country of origin among the currently sequenced *S. schleiferi* ssp. *coagulans* isolates.

In addition to cases of related seal isolates, two other clusters of related *S. schleiferi* ssp. *coagulans* are also apparent in the phylogenetic analysis. These were all canine isolates, but in each case these included isolates from distant countries, the USA and the UK in one instance and the USA and the Republic of Korea in the other. This suggests the international dissemination of these clones and such apparent clonal expansion may represent particularly successful or virulent clones that may merit further investigation. Of course, the phylogenetic tree is limited to a rather small number of available genome sequenced isolates and the sequencing of further isolates from different geographical areas, host species, isolation sites, carriage and disease will greatly improve our knowledge of *S. schleiferi* biology. For instance, certain strains of *S. schleiferi* ssp. *coagulans* may show host specificity, as seen among *Staphylococcus aureus* lineages [40, 41].

None of the isolates showed phenotypic or genotypic resistance to antimicrobials, although it is worth noting that methicillin-resistant *S. aureus* (MRSA) has been isolated from wild harbour seals (*Phoca vitulina*) previously, including one animal inhabiting Scottish waters [42, 43].

In conclusion, we report the isolation and genome sequencing of six *S. schleiferi* ssp. *coagulans* isolates from three species of seal, including Antarctic fur seal, which represents the first report from an otariid species, and the first report from grey seal, which represents the only isolation from a species of seal resident in the Northern Hemisphere to date. We present the first genome-based phylogeny for the *S. schleiferi* species, showing that the two currently recognized subspecies are clearly defined. In addition, while this phylogenetic analysis is limited by the availability of sequenced isolates, it does highlight the international dissemination of canine isolates, which may be linked to the clonal expansion of particularly fit or virulent lineages and should be investigated further.

## Supplementary Data

Supplementary material 1
